# A Study of Abnormal Echocardiogram Findings in Patients With Chronic Kidney Disease With Reference to Cardiac Biomarkers

**DOI:** 10.7759/cureus.65398

**Published:** 2024-07-25

**Authors:** Prakash Shende, Avani Reddy, Vikram B Vikhe, Rahul S Patil, Ahsan A Faruqi, Devansh Khandol

**Affiliations:** 1 General Medicine, Dr. D. Y. Patil Medical College, Hospital and Research Centre, Dr. D. Y. Patil Vidyapeeth (Deemed to be University), Pune, IND

**Keywords:** nt-probnp, cardiovascular disease (cvd), coronary artery disease (cad), chronic kidney disease, troponin i (trop i), left ventricular hypertrophy (lvh), cardiac biomarkers

## Abstract

Background

Chronic kidney disease (CKD) is prevalent, especially in populations with multiple risk factors, such as undiagnosed and untreated hypertension and diabetes mellitus. Cardiovascular diseases (CVDs) leading to poor quality of life or even death have been noted as an increasing trend among CKD patients. This study aims to use cardiac biomarkers to evaluate their association with abnormal echocardiogram findings in CKD patients, which may allow for the improvement of quality of life with early treatment.

Methods and materials

This observational, cross-sectional study was conducted on 103 diagnosed CKD patients at the Department of Medicine, Dr. D.Y. Patil Medical College, Hospital, and Research Centre in Pimpri, Pune, from January 2023 to January 2024. Ethical approval was acquired, and written consent was obtained from participants. The study utilised cardiac biomarkers such as N-terminal pro-B type natriuretic peptide (NT-proBNP), troponin I (Trop I), and a radiological tool, transthoracic echocardiography (TTE). All patients with diagnosed stages 3, 4, and 5 CKD between the ages of 18-80 years were included, and the exclusion criteria consisted of patients who had already undergone cardiac interventional procedures or known cases of CVDs.

Results

In our study, out of 103 participants, the majority were aged between 51 and 60 years (35, 34%). The study had a majority of male participants (76, 73.8%). Major risk factors were considered, noting hypertension in 63 (61.2%) and diabetes mellitus in 81 (78.6%). Participants were divided into stages of CKD. Cardiac biomarkers such as NT-proBNP and Trop I levels were assessed in all participants in the different stages of CKD showing elevated levels of NT-proBNP across all stages. Transthoracic echocardiogram (TTE) screening tests were also evaluated for all patients, showing diastolic dysfunction (DD) as the most common finding in stage 3 (5, 41.67%), stage 4 (25, 62.5%), and stage 5 (35, 68.83%), followed by left ventricular hypertrophy (LVH) as a common finding in stage 3 (4, 33.3%), stage 4 (20, 50%), and stage 5 (30, 58.2%) CKD. Furthermore, the association between raised cardiac biomarkers and abnormal echocardiogram findings across the stages of CKD was evaluated, resulting in a statistically significant association with p-values < 0.05.

Conclusion

This research sheds light on the association between cardiac biomarkers and abnormal echocardiogram findings in CKD patients and helps us determine if there is any added benefit or predictive value in screening these individuals at different stages of the disease to allow early intervention and improvement in treatment and quality of life.

## Introduction

Individuals who suffer from chronic kidney disease (CKD) are more likely to experience cardiovascular complications, such as arrhythmias, heart failure, coronary artery disease and sudden cardiac death. While patients in the early stages of CKD already have a significantly greater incidence and prevalence of cardiovascular events when compared to the general population, individuals with advanced stages of CKD (CKD stages 4-5) show a significantly higher risk. In this high-risk population, cardiovascular disease (CVD) is the primary cause of death rather than kidney failure [1].

To counteract the increased fluid volume brought on by decreased renal function, the cardiac ventricles quickly produce brain natriuretic peptide (BNP) in conjunction with N-terminal pro-brain natriuretic peptide (NT-proBNP) to promote vasodilation and renal output of sodium and water. In addition to compromised cardiac activity, declining renal function (progressive kidney disease) may result in elevated amounts of BNP and NT-proBNP due to increasing intravascular volume. Consequently, different levels of cardiac performance must be included in the appropriate investigation of the impact of renal failure on BNP and NT-proBNP concentration [2]. Elevated troponin I (trop I) levels and NT-proBNP, indicative of myocardial injury and increased wall stress, are commonly seen in patients with CKD. These biomarkers are linked to poor prognoses in CKD, particularly concerning heart failure-related outcomes [3].

CKD populations have high rates of classic CVD risk factors, including diabetes, obesity, dyslipidemia, and hypertension. Left ventricular hypertrophy (LVH) is the most common abnormality in the cardiovascular system. An echocardiogram can be used to assess the mass and volume of the ventricles and is highly accurate in identifying hypertrophy, defining the concentric or eccentric pattern of the ventricle, and measuring the systolic function [4]. Etiological factors contributing to LVH should also be our primary target of management, to curb the incidence, as it is linked to a poor prognosis in CKD patients.

Aims and objectives

This study aims to investigate the abnormal echocardiogram findings seen in patients with CKD using transthoracic echocardiogram (TTE) as a diagnostic tool and the association of these findings with the levels of cardiac biomarkers at different stages of CKD.

## Materials and methods

Study design

This observational, cross-sectional study was conducted at Dr. D. Y. Patil Medical College, Hospital and Research Centre, a tertiary health care centre in Pune, India, between January 2023 and January 2024. The Institutional Ethics Sub-Committee approval was obtained before the commencement of the study (ethical committee clearance number: IESC/PGS/2022/20). Participants were provided with written consent forms to ensure they were aware of the methods, possible risks, and goals of the study. Each participant was evaluated using the appropriate clinical, laboratory, and radiological investigations

Inclusion and exclusion criteria

The inclusion criteria encompassed all known cases of CKD patients who were aged between 18 and 80 years with no prior history of CVD, while the exclusion criteria had patients below the age of 18 years or above the age of 80 years, those with any prior history of CVD, any history of interventional cardiac procedures, or those classified as stage 1 or 2 of CKD.

Sample size

Considering the proportion of patients with elevated cardiac biomarkers among CKD patients is 39.6% from the study by Sommerer et al. [5], with a confidence interval of 95% CI and an acceptable difference of 10% with attrition of 10%, the sample size calculated is 103. The software used is WinPepi version 11.38 (J. H Abramson, Brixton Health, United Kingdom).

Data collection and consent

A detailed clinical history was taken to determine comorbidities, such as hypertension and diabetes mellitus, addiction history, and CKD history, including the history of hemodialysis. Necessary blood tests such as renal function tests and urine for microalbuminuria were assessed. Individuals were classified into stages 3, 4, and 5 of CKD using estimated glomerular filtration rate (eGFR) categories (ml/min/1.73m²) and levels of albuminuria, according to the Kidney Disease Improving Global Outcomes (KDIGO) 2024 nomenclature [6]. Cardiac biomarkers NT-proBNP and Trop I were assessed for all participants, alongside TTE, to evaluate various abnormal echocardiogram findings, their prevalence in our study, and the significance of their association. Strict adherence to informed consent and ethical approval procedures guaranteed conformity with institutional and global norms. The gathered information was carefully examined to determine the results of our study.

Statistical analysis

Descriptive statistics were expressed as mean values ± standard deviations for continuous variables. Categorical variables were expressed as proportions, and the association in proportions was investigated using the chi-square test. Statistical significance was defined as a P-value of less than or equal to 0.05. Data tracking was performed using Microsoft Excel (Microsoft Corporation, Redmond, WA, USA) and analysed using IBM Statistical Package for the Social Sciences (SPSS) Statistics (Version 26.0, Chicago, IL: IBM Corp).

## Results

**Figure 1:**
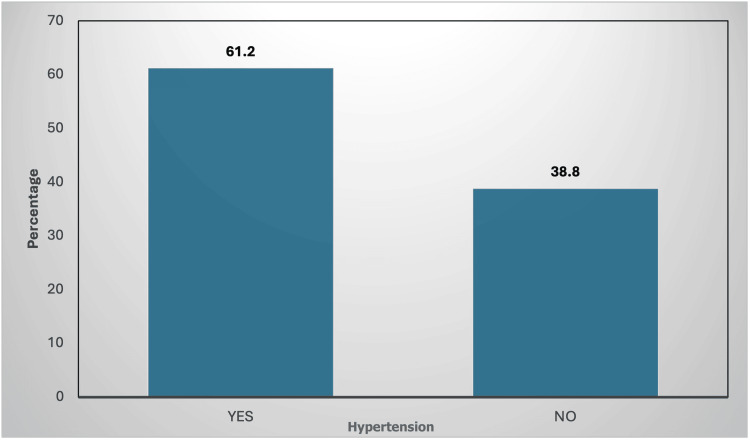
Distribution of systemic hypertension among the study participants (n = 103) n, total number of subjects; Yes, are hypertensive; No, are not hypertensive Figure credit: Avani Reddy

**Figure 2:**
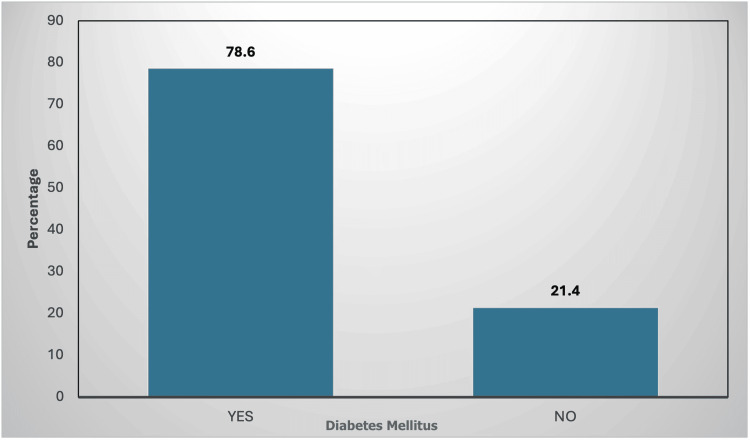
Distribution of diabetes mellitus among the study participants Yes, have diabetes; No, are not diabetic Figure credit: Avani Reddy

In our study, out of 103 participants, the majority were aged between 51 and 60 years (35, 34%), followed by 41-50 years (33, 32%), 61-70 (23, 22.3%), 31-40 (6, 5.8%), 20-30 (3, 2.9%), and 71-80 (3, 2.9%) (Table 1). The study had male (76, 73.8%) and female participants (27, 26.2%) (Table 2). Major risk factors were considered, noting hypertension in 63 (61.2%) and non-hypertensives in 40 (38.8%) (Figure 1) and diabetes mellitus in 81 (78.6%) and non-diabetics in 22 (21.4%) (Figure 2).

**Table 1:** Age distribution of the participants N, number of subjects

Age (years)	N (%)
20-30	3 (2.9)
31-40	6 (5.8)
41-50	33 (32)
51-60	35 (34)
61-70	23 (22.3)
71-80	3 (2.9)

**Table 2:** Gender distribution among the participants

Gender	N (%)
Male	76 (73.8)
Female	27 (26.2)

**Figure 3:**
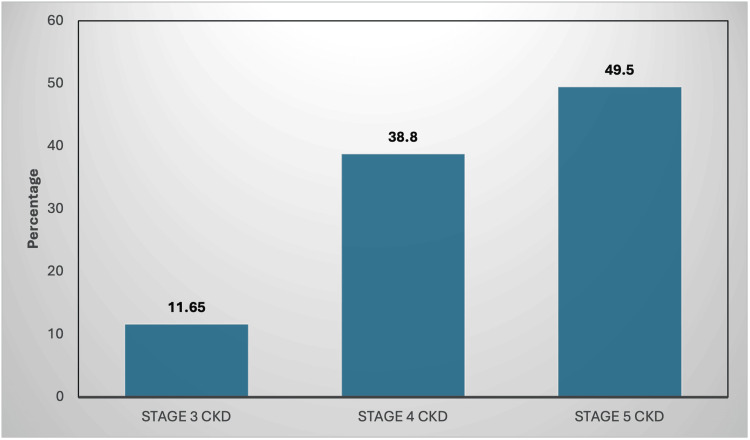
Distribution according to the stage of CKD among the study participants (n = 103) CKD, chronic kidney disease Figure credit: Avani Reddy

The total number of subjects (n = 103) were categorised according to the stage of CKD, showing 51 (49.5%) subjects in stage 5 of CKD, 40 (38.8%) in stage 4, and 12 (11.65%) subjects in 3 (Figure 3).

Stage 3 CKD showed that the highest mean NT-Pro BNP level is 1336.34 ± 389.37 pg/mL, followed by stage 4 with 1253.57 ± 516.06 pg/ml and stage 5 with 1268 ± 169 pg/ml. Trop I (ng/mL) levels were noted as 0.05 ± 0.01 in stage 3, 0.07 ± 0.03 in stage 4, and 0.25 ± 0.03 in stage 5 (Table 3). These levels of NT proBNP indicate persistent cardiac stress throughout CKD progression. Slightly elevated Trop I levels were noted in the study, which may suggest increased myocardial damage in CKD patients.

**Table 3:** Levels of cardiac biomarkers in different stages of CKD in the study participants The data are represented as mean ± standard deviation. n, number of subjects in that particular stage of CKD, pg/ml, picograms per millilitre, ng/ml, nanograms per millilitre

Cardiac biomarkers	Normal values	Stage 3 CKD (n = 12) (mean ± standard deviation)	Stage 4 CKD (n = 40) (mean ± standard deviation)	Stage 5 CKD (n = 51) (mean ± standard deviation)
NT ProBNP (pg/mL)	<450	1336.34 ± 389.37	1253.57 ± 516.06	1268 ± 169
TROP-I(ng/mL)	0-0.4	0.05 ± 0.01	0.07 ± 0.03	0.25 ± 0.03

**Figure 4:**
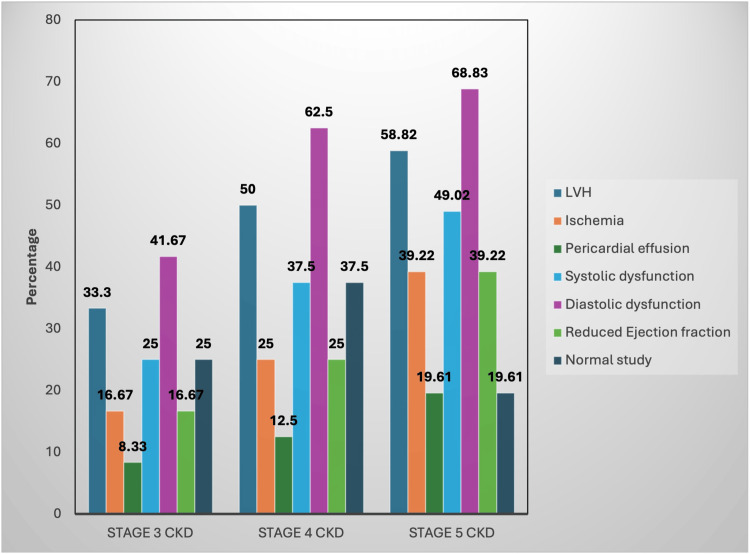
Distribution of TTE findings among the study participants across the different stages of CKD (n = 103) Represented as the affected percentage of the total subjects per group of the respective stage of CKD. TTE, transthoracic echocardiography; CKD: chronic kidney disease Figure credit: Avani Reddy

According to the results of TTE screenings of all study participants, the highest cardiovascular abnormality found was diastolic dysfunction (DD) noted in stage 3 with five cases (41.67%), in stage 4 with 25 cases (62.5%), in stage 5 with 35 cases (68.83%), followed by LVH as a common finding with four cases (33.3%), 20 cases (50%), and 30 cases (58.2%); systolic dysfunction with three cases (25%), 15 cases (37.5%), and 25 cases (49.02%); ischemia and reduced ejection fraction with two cases (16.67%), 10 cases (25%), and 20 cases (39.22%); and pericardial effusion with one case (8.33%), five cases (12.5%), and 10 cases (19.61%) noted in stage 3, 4, and 5 CKD, respectively. Normal studies were noted in CKD stage 3 with three cases (25%), stage 4 with 15 cases (37.5%), and stage 5 with 10 cases (19.61%) (Figure 4).

We assessed our results to evaluate statistical significance using p-values. Accordingly, we obtained p-values of 0.007, <0.001, and <0.001 in stages 3, 4, and 5 CKD, proving that there was a significant association between raised levels of cardiac biomarkers and cardiovascular abnormalities observed in TTE in subjects across all stages of CKD (Table 4).

**Table 4:** Association of elevated cardiac biomarkers and any abnormal findings in TTE in participants across all stages of CKD n, the total number of subjects per stage of CKD. A p-value of <0.05 is considered statistically significant.

Stage of CKD	Number of participants (n)	Subjects with elevated levels of cardiac biomarkers	Subjects with any abnormal findings in TTE	p-value
Stage 3	12	9	9	0.007
Stage 4	40	28	25	<0.001
Stage 5	51	38	41	<0.001

## Discussion

In our study, out of 103 participants, the majority were aged between 51 and 60 years (35, 34%), followed by 61-70 years (23, 22.3%). The study had a majority of male participants (76, 73.8%). In our study, significant risk factors identified were hypertension (63, 61.2%) and diabetes mellitus (81, 78.6%). This finding aligns with the broader understanding that the increase in cardiovascular events with CKD progression is primarily due to the interplay of traditional and non-traditional cardiovascular risk factors. Traditional factors, extensively documented in the Framingham study, include age, male gender, hypertension, diabetes mellitus, hyperlipidaemia, and smoking [7].

Our research classified individuals into different stages of CKD and assessed for elevated cardiac biomarkers and their correlation with abnormalities detected through TTE. DD was the most prevalent cardiovascular abnormality, found in stage 3 (5, 41.67%), stage 4 (25, 62.5%), and stage 5 (35, 68.83%), followed by LVH in stage 3 (4, 33.3%), stage 4 (20, 50%), and stage 5 (30, 58.2%). These findings align with a study by Shaikh et al., which reported that 73% of patients with elevated NT-proBNP had LVH, and 69% with elevated Trop I exhibited signs of DD [8].

Importantly, our results showed a significant positive correlation between elevated levels of NT-proBNP and abnormal TTE findings across all stages of CKD, with p-values of 0.007, <0.001, and <0.001 for stages 3, 4, and 5, respectively. This is consistent with the multicentre, prospective cohort study by Wada et al., which involved 3,255 patients with suspected or confirmed coronary artery disease (CAD). In their research, NT-proBNP was significantly linked to three-point major adverse cardiovascular events (3P-MACE), which included cardiovascular death, nonfatal myocardial infarction, and nonfatal stroke, as well as five-point major adverse cardiovascular events (5P-MACE), which added heart failure hospitalisation and revascularisation procedures [9]. In addition, David et al. found that 65% of CKD patients with elevated NT-proBNP levels had LVH. Their study demonstrated that NT-proBNP effectively diagnosed left ventricular dysfunction (LVD) with a cut-off value of 7168 ng/L [10]. Importantly, despite confounding factors such as volume overload and reduced renal excretion, NT-proBNP remains a valuable biomarker for detecting cardiac structural changes. Together, these studies underscore the significance of NT-proBNP in assessing abnormal cardiac findings in CKD patients.

Cardiovascular-related reasons account for an estimated 40-50% of all deaths in patients with CKD stages 4 and 5, whereas this percentage is just 26% in people with normal kidney function [11]. A study conducted by Dietl et al. from the KORA platform in Germany evaluated the association between NT-proBNP levels and cardiovascular mortality, independent of clinical cardiac remodelling measured by echocardiography. The study involved 1,223 participants and found that higher NT-proBNP levels were significantly associated with increased mortality risk. Specifically, individuals with elevated NT-proBNP levels had a 30% higher risk of cardiovascular mortality, even after adjusting for LVH, DD, and ejection fraction [12]. A large, diversified, real-world population study, conducted by Yu A et al., showed that 18.6% of patients with severe CKD and an eGFR of less than 45 mL/min/1.73 m² had prevalent heart failure with preserved ejection fraction (HFpEF). Compared to patients without heart failure, the age and sex-adjusted one-year death rate was 11 times greater in CKD patients with heart failure [13].

As shown in previous studies, there is an increased incidence of mortality in CKD patients with cardiovascular abnormalities, our primary goal should be to curb this incidence using tools like cardiac biomarkers. In a cohort study by Oka et al. involving 2,998 patients with non-dialysis CKD, monitoring BNP levels was linked to a reduced risk of kidney replacement therapy, acute kidney injury, and hospitalisation due to heart failure. These findings suggest that BNP monitoring can help physicians manage fluid levels more effectively, potentially leading to better kidney outcomes. Overall, this underscores the importance of BNP monitoring in enhancing cardiovascular and kidney health in CKD patients [14].

A meta-analysis and systematic review by Su et al. demonstrated a 15% reduction in the likelihood of significant cardiovascular events in CKD patients treated with antiplatelet medication. Their analysis indicated an overall net benefit, suggesting that the potential risk of bleeding is outweighed by the substantial prevention of cardiovascular events with antiplatelet drugs. Nevertheless, careful monitoring and individualised assessment are always essential [15]. For individuals with CAD, medical treatment remains the cornerstone of care, significantly enhancing survival and quality of life. In patients with both CAD and CKD, the effective management of classic risk factors, such as diabetes, dyslipidaemia, and hypertension, is crucial. Proper management can prevent adverse cardiovascular outcomes and slow the progression of CKD [16]. Effective treatment strategies for CKD with CVD necessitate a comprehensive approach that addresses both conditions simultaneously.

Limitations

When interpreting the findings, it is important to consider the limitations of the current study. We acknowledge that the study was conducted only in a single centre with a restricted study population. More extensive research is required to validate current findings. Even if the results were corrected, residual confounding variables may still have an impact on plasma NT-proBNP levels and patient outcomes in the study group. Due to financial constraints, the current study did not include a serial assessment of NT-proBNP levels, which could have helped identify treatment failure and modify it promptly.

## Conclusions

Keeping in mind the results of the study and the comparison and knowledge gained from other studies, it goes without saying that cardiac biomarkers ought to be screened in CKD patients as part of a routine assessment. This baseline screening followed by consecutive tests and radiological investigations will allow us to predict cardiovascular complications in advance and enable early treatment, which may improve the quality of life of these patients. Integrating NT-proBNP monitoring with the management of traditional risk factors such as hypertension and diabetes is crucial. Despite our study's limitations, the evidence supports a comprehensive approach to cardiovascular care in CKD patients, aiming to reduce mortality and improve overall outcomes.
